# Comparison of the prevalence of incidental and non-incidental papillary thyroid microcarcinoma during 2008–2016: a single-center experience

**DOI:** 10.1186/s12957-018-1501-8

**Published:** 2018-10-10

**Authors:** Krzysztof Kaliszewski, Agnieszka Zubkiewicz-Kucharska, Paweł Kiełb, Jerzy Maksymowicz, Aleksander Krawczyk, Otto Krawiec

**Affiliations:** 1 First Department and Clinic of General, Gastroenterological, and Endocrine Surgery, Wroclaw Medical University, 66 Maria Skłodowska-Curie Street, 50-369 Wrocaw, Poland; 2 Department of Endocrinology and Diabetology for Children and Adolescents, Wroclaw Medical University, Wroclaw, Poland

**Keywords:** Incidental, Non-incidental, Papillary thyroid microcarcinoma

## Abstract

**Background:**

The incidence of papillary thyroid microcarcinoma (PTMC) is increasing; however, it is not clear whether this reflects an increase in the incidence of incidental or in that of non-incidentally (presurgically) discovered PTMC (IPTMC vs. NIPTMC). We assessed the incidence of IPTMC and NIPTMC over the past 9 years, to discern whether the increase in PTMC incidence is due to improved diagnostics or to a real increase in the incidence.

**Methods:**

We performed a retrospective chart review of 4327 patients who were consecutively admitted to and surgically treated for thyroid pathology at a single institution. As a main presurgical diagnostic test, all patients underwent ultrasound-guided fine-needle aspiration biopsy (UG-FNAB). The analyzed time frame was divided into three equal periods (I: 2008–2010, II: 2011–2013, III: 2014–2016), and IPTMCs and NIPTMCs were assessed and compared in each period.

**Results:**

We evaluated 393 (9.08%) patients with thyroid malignancy, of which 156 (3.60% of all thyroid tumors [TTs]; 39.69% of all thyroid cancers [TCs]) were diagnosed as PTMC. The prevalence of NIPTMC among all TCs increased from 16.66% in 2008 to 33.75% in 2016, while that of IPTMC decreased from 20.83% in 2008 to 13.75% in 2016. The incidence rates of NIPTMC and IPTMC in period III differed statistically significantly (*p* < 0.0001). The prevalence rate of NIPTMC in period III was higher than that in period II, yet comparable to that in period I (*p* = 0.0014; *p* = 0.2804, respectively).

**Conclusions:**

The prevalence of NIPTMC, rather than that of IPTMC, is escalating; this may be due to better presurgical diagnosis.

## Background

Thyroid carcinoma (TC) is the most common malignant tumor of the endocrine system [1]. It constitutes approximately 1% of all human malignancies and is the main cause of death among endocrine tumor-related deaths [2]. In 2010, Jemal et al. reported 44,700 new cases of thyroid cancers per year, worldwide, and 1700 deaths due to this condition occurred annually [3]. An annual increase of 5.3% in TC incidence was reported by Magreni et al. in 2015 [4].

Papillary thyroid cancer (PTC), which is the main type of TC, accounts for 80% of all thyroid malignancies [5]. The prognosis of PTC is generally favorable; however, for some subtypes, prognosis may depend on the cancer stage at the time of diagnosis. For early stages, the 10-year survival rate reaches 90%, whereas for later stages, the 10-year survival rate is not as high [5].

A clinically important type of PTC is a tumor with a small size (diameter ≤ 1.0 cm), regardless of whether lymph node and local invasion, as well as distant metastases, has occurred. The World Health Organization (WHO) defines these tumors as a papillary thyroid microcarcinoma (PTMC) [6]. PTMCs account for approximately 30% of all PTCs [7]. Some authors have described PTMCs as tumors with low malignancy, which are slow-growing, minimally invasive, and associated with low mortality [8]. Based on autopsy studies, Solares et al. have confirmed that up to 36% of PTMCs had low aggressiveness [9]. Other reports have also described these tumors as common and typical findings, with a favorable prognosis [10]. However, very aggressive forms of PTMC have also been described [11]. Therefore, Gao et al. stated that PTCs with a small size (≤ 1.0 cm in diameters) do not always behave as indolent tumors [12].

To date, the diagnosis of PTMC has been highly reliant on a high-frequency ultrasonography examination and ultrasound-guided fine-needle aspiration biopsy (UG-FNAB), which remains the standard diagnostic procedure for the evaluation of thyroid nodules. The main purpose of UG-FNAB is to distinguish between malignant and benign tumors and to identify patients requiring surgical treatment [13].

The accuracy of diagnostic procedures for thyroid nodules has improved over the last few years, and the ease of access to such diagnostic modalities may be the reason for the higher prevalence of thyroid tumors. However, the exact cause of this increase is still debated. As we have observed a continuous increase in the PTMC incidence over a number of years, we have contemplated various reasons for this phenomenon. Although improvements in the imaging tools, availability of UG-FNAB, and easier access to diagnostic thyroid pathology may play a role, some changes in the environment may also have caused a real increase in the morbidity rate.

The purpose of this study was to assess and compare the incidence of PTMC over the last 9 years and to identify whether there is a difference in the number of presurgically discovered (non-incidental) and non-discovered (incidental) PTMCs, to determine whether the increased incidence in PTMC is due to improved diagnostics (mainly UG-FNAB).

## Methods

We performed a retrospective chart review of 4327 patients who were consecutively admitted to and surgically treated for thyroid pathology at a single institution, from January 1, 2008, to December 31, 2016. The analyzed time frame was divided into three equal periods (period I: 2008–2010, period II: 2011–2013, period III: 2014–2016). Next, we assessed and compared the incidence rates of incidental PTMCs (IPTMCs) and non-incidental PTMCs (NIPTMCs) in these three periods. All of the ultrasound examinations were performed by the same team of radiologists experienced in thyroid sonography. All patients underwent UG-FNAB as the main presurgical diagnostic test. The same equipment and ultrasonography set in all biopsies were used. A 10-MHz linear probe of ultrasonography set has been applied. The UG-FNAB was performed using 0.5-mm gauge needles and 10-cc syringes for each procedure. Clinical and pathological classification was performed according to the TNM classification criteria (7th Edition, 2015) by the American Joint Committee on Cancer (AJCC) [14]. All of the patients underwent total thyroidectomy by the same team of surgeons experienced in thyroid surgery, and all histopathological specimens were examined by the same two pathologists, who were both experienced in diagnosing thyroid malignancy.

### Statistical analysis

Statistical analysis was conducted with the use of Statistica vs. 12 (StatSoft, Inc., Tulsa, OK, USA; 2014). The following statistical measures were used: arithmetical mean (x), median and standard deviation (SD), and ranges of determined parameters in study groups.

The Shapiro-Wilk test was used to confirm the normality of data distribution. As data demonstrated a normal distribution, *t* tests were used to assess the significance of differences. Intergroup frequency assessment was performed using a chi-squared test. Yate’s correction was applied when the expected frequency was less than 5 or the total count was less than 50.

*P* values < 0.05 were taken as indicating statistically significant differences, while *p* values from 0.05 to < 0.10 were considered as indicating borderline statistical significance.

## Results

From 4327 patients diagnosed for thyroid pathology at our institute during the study period, we evaluated 393 (9.08%) patients with thyroid malignancy, of whom 156 (3.60% of total thyroid pathology; 39.69% of TC patients) were diagnosed with PTMC. In this homogenous group, there were 52 (33.33%) patients with IPTMC and 104 (66.67%) with NIPTMC. There were 45 (89.18%) females and 7 (10.82%) males in the IPTMC group and 98 (95%) females and 6 (5%) males in the NIPTMC group (*p* = 0.1013). The mean age of all patients with PTMC was 48.6 (± 14.6), and 44.6 (± 15.9) and 49.0 (± 14.5) for males and females, respectively (*p* = 0.1390; Table 1).

**Table 1:** Demographic characteristics of patients with a diagnosis of papillary thyroid microcarcinoma (PTMC)

Parameter	PTMC (*n* = 156)
Gender	
Male	13 (8.3%)
Female	143 (91.7%)
Age (years)	
All patients	48.6 ± 14.6
Male	44.6 ± 15.9
Female	49.0 ± 14.5

Descriptive data are presented as numbers (*n*), percentage (%) and mean ± standard deviation (± SD)

**Fig. 1:**
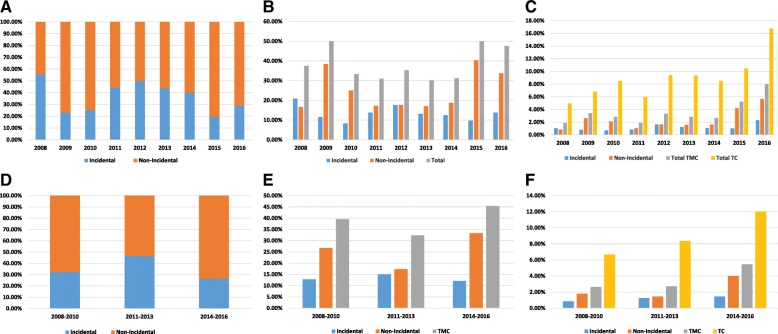
**a** The percentage of incidental and non-incidental papillary thyroid microcarcinomas in the period 2008–2016. **b** The percentage of incidental and non-incidental papillary thyroid microcarcinomas, and all papillary thyroid microcarcinomas according to total number of thyroid carcinomas in the period 2008–2016. **c** The percentage of incidental and non-incidental papillary thyroid microcarcinomas, total papillary thyroid microcarcinomas, and total thyroid carcinomas according to total number of thyroid tumors in the period 2008–2016. **d** The percentage of incidental and non-incidental papillary thyroid microcarcinomas in three equal time-periods. **e** The percentage of incidental, non-incidental papillary thyroid microcarcinomas, and total papillary thyroid microcarcinomas according to total number of thyroid carcinomas in three equal time-periods. **f** The percentage of incidental and non-incidental papillary thyroid microcarcinomas, total papillary thyroid microcarcinomas, and total thyroid carcinomas according to total number of thyroid tumors in three equal time-periods. TMC, thyroid microcarcinoma; TC, thyroid carcinoma

The prevalence of PTMC increased over the 9-year period, from 37.5% in 2008 to 47.5% in 2016 (*p* = 0.0414). The prevalence of NIPTMC increased from 16.66% of all TCs in 2008 to 33.75% in 2016, but IPTMC decreased from 20.83% in 2008 to 13.75% in 2016 (Table 2). In the years 2009, 2015, and 2016, we observed more patients with NIPTM than with IPTMC (Table 3; Fig. 1a, b and c). Moreover, we noticed a statistically significant predominance of NIPTMC over IPTMC in periods I (2008–2010) and III (2014–2016), but not in period II (2011–2013), (*p* = 0.0216; *p* < 0.0001; *p* = 0.6176. respectively; Table 4, Fig. 1d, e, and f). Furthermore, there was an increase in the prevalence of NIPTMC in period III as compared to period II (*p* = 0.0014), but not as compared to period I (*p* = 0.2804) (Table 5).

**Table 2:** The prevalence of incidental and non-incidental papillary thyroid microcarcinoma (IPTMC and NIPTMC) according to all thyroid tumors and all thyroid cancers in years 2008–2016

	2008	2009	2010	2011	2012	2013	2014	2015	2016
For all thyroid tumors									
IPTMC	5 (1.03%)	3 (0.79%)	3 (0.71%)	4 (0.83%)	9 (1.66%)	7 (1.24%)	4 (1.06%)	6 (1.01%)	11 (2.31%)
NIPTMC	4 (0.83%)	10 (2.62%)	9 (2.13%)	5 (1.04%)	9 (1.66%)	9 (1.59%)	6 (1.60%)	25 (4.21%)	27 (5.66%)
PTMC	9 (1.86%)	13 (3.40%)	12 (2.84%)	9 (1.86%)	18 (3.31%)	16 (2.83%)	10 (2.66%)	31 (5.22%)	38 (7.97%)
All thyroid cancers	24 (4.96%)	26 (6.81%)	36 (8.51%)	29 (6.00%)	51 (9.39%)	53 (9.38%)	32 (8.51%)	62 (10.44%)	80 (16.77%)
All thyroid tumors	484 (100%)	382 (100%)	423 (100%)	483 (100%)	543 (100%)	565 (100%)	376 (100%)	594 (100%)	477 (100%)
For all thyroid cancers									
IPTMC	5 (20.83%)	3 (11.53%)	3 (8.33%)	4 (13.79%)	9 (17.64%)	7 (13.20%)	4 (12.5%)	6 (9.67%)	11 (13.75%)
NIPTMC	4 (16.66%)	10 (38.46%)	9 (25%)	5 (17.24%)	9 (17.64%)	9 (16.98%)	6 (18.75%)	25 (40.32%)	27 (33.75%)
PTMC	9 (37.5%)	13 (50%)	12 (33.33%)	9 (31.03%)	18 (35.29%)	16 (30.18%)	10 (31.25%)	31 (50%)	38 (47.5%)
All thyroid cancers	24 (100%)	26 (100%)	36 (100%)	29 (100%)	51 (100%)	53 (100%)	32 (100%)	62 (100%)	80 (100%)

*IPTMC* incidental papillary thyroid microcarcinoma, *NIPTMC* non-incidental papillary thyroid microcarcinoma, *PTMC* papillary thyroid microcarcinoma

**Table 3:** The prevalence of incidental and non-incidental papillary thyroid microcarcinoma in each year

	Incidental	Non-incidental	Total	*p*
2008	5 (1.03%)	4 (0.83%)	9 (1.86%)	0.7115
2009	3 (0.79%)	10 (2.62%)	13 (3.40%)	*0.0261*
2010	3 (0.71%)	9 (2.13%)	12 (2.84%)	0.0578
2011	4 (0.83%)	5 (1.04%)	9 (1.86%)	0.7169
2012	9 (1.66%)	9 (1.66%)	18 (3.31%)	1
2013	7 (1.24%)	9 (1.59%)	16 (2.83%)	0.5874
2014	4 (1.06%)	6 (1.60%)	10 (2.66%)	0.4911
2015	6 (1.01%)	25 (4.21%)	31 (5.22%)	*0.0001*
2016	11 (2.31%)	27 (5.66%)	38 (7.97%)	*0.003*

**S**tatistically significant differences are shown in italics

**Table 4:** Patients with incidental and non-incidental papillary thyroid microcarcinoma, all thyroid cancers and all papillary thyroid microcarcinoma cases in three equal time-periods

NIPTMC	52 (33.3%)		
IPTMC	104 (66.7%)		
	Period I (2008–2010)	Period II (2011–2013)	Period III (2014–2016)
Number of cases in each period [*n*]			
NIPTMC	23	23	58
IPTMC	11	20	21
TC	86	133	174
Percentage of PTMC in all TC cases in each period [%]			
NIPTMC	26.74%	17.29%	33.33%
IPTMC	12.79%	15.04%	12.07%
PTMC	39.53%	32.33%	45.40%
*p*	*0.0216*	0.6176	*< 0.0001*

Statistically significant differences are shown in italics

*IPTMC* incidental papillary thyroid microcarcinoma, *NIPTMC* non-incidental papillary thyroid microcarcinoma, *PTMC* papillary thyroid microcarcinoma, *TC* all thyroid cancers

**Table 5:** Comparison of the three equal periods of time according to incidence of non-incidental and incidental papillary thyroid microcarcinoma

	*p*		
	Period I (2008–2010)	Period II (2011–2013)	Period III (2014–2016)
Period I (2008–2010)	–	0.0882	0.2804
Period II (2011–2013)	0.0882	–	*0.0014*
Period III (2014–2016)	0.2804	*0.0014*	–

Statistically significant differences are shown in italics

## Discussion

The incidence of PTMC has been increasing worldwide. In our study, we observed that the prevalence of this tumor has increased more than four times, from 1.86% of all thyroid tumors in 2008 to 7.97% in 2016. Although it is typically minimally invasive, Gao et al. described PTMC as a public health concern because of its tremendous increase in the past few decades [12]. Additionally, it has been suggested that if any additional factors, such as extrathyroidal invasion, lymph node metastases, or the *BRAF* V600E mutation, are found, PTMC should be treated as a “larger” papillary thyroid cancer [15].

Postoperatively, PTMC is often found in multinodular goiter (MNG) and it is then diagnosed as IPTMC [16]. In our study, the prevalence of IPTMC increased from 1.03% of all thyroid tumors in 2008 to 2.31% in 2016, together with the increase of all PTMCs (from 1.86% in 2008 to 7.97% in 2016). In terms of total PTMC, the prevalence of IPTMC has decreased approximately by half: from 20.83% in 2008 to 13.75% in 2016. Li et al. reported that IPTMC is undetectable before surgery, due to its coexistence with MNG, its small size, and its deep localization within thyroid gland [17]. For that reason, in MNG, every nodule should be assessed by ultrasound examination to determine the risk of cancer. In our previous study, we revealed that the assistance of a radiologist in UG-FNAB procedures increases the value of the procedure [18].

It was suggested that pre-operative diagnosis of PTMC is difficult and therefore rare, because of its slow growth rate, absence of specific symptoms, clinical characteristics, and potential co-occurrence with benign thyroid nodules [19]. Some authors have stated that the increasing rate of the prevalence of non-incidental thyroid microcarcinoma (NIPTMC) during the past few decades may have been due to the extensive development of high-frequency ultrasonography and the UG-FNAB technique [20]. These observations are in accordance with the results of our study. At the beginning of this trial, in 2008, only 0.83% of all thyroid tumors were NIPTMC, whereas this figure was 5.66% in 2016.

The mortality rate of PTMC has remained unchanged over the last few decades, which additionally supports the hypothesis of increased NIPTMC diagnoses and treatment [21]. At present, even tumors with a 3-mm diameter can be detected by ultrasonography and subsequently qualify for UG-FNAB [15]. For this reason, a large number of very small malignant tumors are found, thus increasing the rate of NIPTMC. Chen et al. suggested that even nodules with a dimension of 2–3 mm can be detected with the use of a high-resolution transducer [19]. Comparing the rates of NIPTMC to those of IPTMC in the equally divided time-periods of our study, we noticed a statistically significant increase of NIPTMC rate in the last period. In those years, even very small tumors with suspicions of malignancy qualified for UG-FNAB, followed by early radical surgery [16]; consequently, the “wait and see” approach until the tumor had increased in size was rejected [22, 23]. We propose that this is the reason for the continuous and significant increase in NIPTMC diagnoses. All of the patients admitted to our clinic for thyroid tumors, who subsequently underwent surgical treatment, also underwent UG-FNAB. This diagnostic method was widely used to distinguish PTMC from benign tumors. Nevertheless, not every PTMC diagnosed postoperatively had been biopsied before surgical treatment; thus, not every “suspicious” tumor was selected for biopsy. This observation explains the fact that, even in period III (2014–2016), in which high-quality UG-FNAB was available, we still observed 12.07% of IPTMC. This phenomenon may also be explained by the observation of Brito et al. [21], who noticed that increasing rates of thyroid operations were coupled with more radical surgical treatment. We used a more radical treatment strategy, and it may justify the high incidence rate of IPTMC [21]. Castro et al. [24] concluded that the major reason for the increasing incidence of NIPTMC is the detection of subclinical, indolent tumors, thus, overdiagnosis. Regarding the indolent type of NIPTMC, it has been suggested to change the term “carcinoma” to “small papillary lesions.” [24] This might be more suitable for patients in terms of the overdiagnosis and overtreatment phenomenon.

Our study has certain limitations. Firstly, this was a retrospective study and included patients who underwent UG-FNAB and thyroid surgery during a relatively not very long time. Secondly, we could not perform lymph nodes status analysis, because of retrospective study design. Patients with IPTMC did not received central lymph node dissection, because the cancer diagnosis was established postsurgery. And finally, the study included a relatively small number of patients. Unfortunately, surgery still not only represents a treatment option but also is a diagnostic tool in thyroid pathology. Microscopic evaluation of the surgical specimen still represents the gold standard for the diagnosis of thyroid nodules. In regards to overdiagnosis and overtreatment, further studies are necessary to resolve these issues.

## Conclusions

The proportion of IPTMC and NIPTMC of all thyroid tumors is relatively high and is increasing. However, in terms of total TCs, only the prevalence of NIPTMC, but not that of IPTMC, is increasing; this may be explained by better presurgical diagnostic processes.

## Abbreviations

IPTMC: Incidental papillary thyroid microcarcinoma
MNG: Multinodular goiter
NIPTMC: Non-incidental papillary thyroid microcarcinoma
PTC: Papillary thyroid cancer
PTMC: Papillary thyroid microcarcinoma
TC: Thyroid cancer
TT: Thyroid tumor
UG-FNAB: Ultrasound-guided fine-needle aspiration biopsy
